# Resilon: A Comprehensive Literature Review

**DOI:** 10.5681/joddd.2013.030

**Published:** 2013-08-30

**Authors:** Mehrdad Lotfi, Negin Ghasemi, Saeed Rahimi, Sepideh Vosoughhosseini, Mohammad Ali Saghiri, Atabak Shahidi

**Affiliations:** ^1^Professor, Department of Endodontic, Faculty of Dentistry, Tabriz University of Medical Sciences, Tabriz, Iran; ^2^Postgraduate Student, Department of Endodontic, Faculty of Dentistry, Tabriz University of Medical Sciences, Tabriz, Iran; ^3^Dental and Periodontal Diseases Research Center, Tabriz University of Medical Sciences, Tabriz, Iran; ^4^Associate Professor, Department of Oral and Maxillofacial Pathology, Faculty of Dentistry, Tabriz University of Medical Sciences, Tabriz, Iran; ^5^Research Associated, Department of Ophthalmology and Visual Sciences, University of Wisconsin School of Medicine and Public health, Madison, WI, USA; ^6^Independent Research Scientist, Orumieh, Iran

**Keywords:** Antibacterial activity, chemical and physical properties, clinical outcome, cytotoxicity, disinfection, leakage, Resilon, retreatment

## Abstract

***Background and aims.*** An ideal root canal filling material should completely seal the entire root canal space and block communication between the root canal system and its surrounding tissues; it should also be nontoxic, noncarcinogenic, non-genotoxic, biocompatible, insoluble in tissue fluids and dimensionally stable. Bonding to dentin is a promising property, which can prevent leakage and improve the sealing ability of root canal filling materials. Resilon was developed and rec-ommended initially because the existing rootcanal filling materials did not bond to root canal dentin. Since its introduction in 2004, numerous reports have been published regarding various aspects of this material. The aim of this literature review is to present investigations regarding Resilon’s physical and chemical properties and leakage studies.

***Materials and methods.*** A review of the literature was performed by using electronic and hand searching methods for Resilon from May 2004 to April 2012.

***Results.*** There are many published reports regarding Resilon. The searchshowed that Resilon is composed of a parent polymer, polycaprolactone or Tone, which is a biodegradable aliphatic polyester, with filler particles consisting of bioactive glass, bismuth oxychloride and barium sulfate. It possesses some antibacterial and antifungal properties. It is a promising material for root canal filling. Despite the presence of numerous case reports and case series regarding these applications, there are few designed research studies on clinical applications of this material. Resilon has some drawbacks such as high cost.

***Conclusion.*** Resilon seals well and is a biocompatible material. However, more clinical studies are needed to confirm its efficacy compared with other materials.

## Introduction

Most endodontic failures occur as a result of leakage of irritants into periapical tissues.^1^ An ideal orthograde filling material should prevent communication between the root canal system and its surrounding tissues. It should also be nontoxic, noncarcinogenic, nongenotoxic, biocompatible with host tissues, insoluble in tissue fluids and dimensionally stable.^2^ Furthermore, the presence of moisture should not affect its sealing ability; it should be easy to use and should be radiopaque for visibility on radiographs.^3^ Because existing root canal filling materials do not possess these ‘‘ideal’’ characteristics, Resilon was developed and recommended as a root canal filling material. Resilon has been recognized as a material that bonds to dentin when Epiphany sealer is used.^4^ The sealing ability of Resilon has been tested by leakage studies and scanning electron microscopy (SEM). Because materials used in endodontics are frequently placed in close contact with the periodontium, they also must be biocompatible with host tissues. Moreover, as recommended by the American Association of Endodontists, the use of a new material should be based on laboratory, biological, and clinical studies. Following these steps systematically paves the way for clinical use of a material in experimental animals and then in humans.

The purpose of this literature review is to update previously published information and present a comprehensive list of articles from May 2004 to April 2012 regarding Resilon’s physical and chemical properties, leakage studies, clinical outcomes, retreatment, cytotoxicity, disinfection and antibacterial activity.

### Inclusion and exclusion criteria

Peer-reviewed journals were searched for articles on Resilon published in English from May 2004 to April 2012. Studies that did not meet these criteria were excluded.

### Search methodology

Appropriate MeSH headings and key words related to different aspects of Resilon were searched in PubMed and Cochrane databases.

Moreover, hand search of the last 2 years of issues of the following endodontic journals was conducted: International Endodontic Journal; Journal of Endodontics; Oral Surgery, Oral Medicine, Oral Pathology, Oral Radiology and Endodontology; Brazilian Journal of Endodontics and Journal of Dental Materials. The process of cross-referencing continued until no new articles were identified.

### Chemical composition and interaction/

Resilon is a thermoplastic synthetic polymer-based root canal filling material composed of a parent polymer, polycaprolactone or Tone, which is a biodegradable aliphatic polyester, with filler particles consisting of bioactive glass, bismuth oxychloride and barium sulfate. Resilon polymeric matrix consists of 25-40% polycaprolactone (PCL) and 3-10% dimethacrylates.^5^ Degradation pattern and ion release of Resilon show that sodium and calcium ions are the only ions released. Visual methods have shown a faint surface residue on the surface of Resilon.^6^ One study examined susceptibility of Resilon and gutta-percha to microbial biodegradation and showed minimal changes in the surface integrity of gutta-percha; however, Resilon exhibited severe surface pitting and erosion.^7^

### Physical properties

**Cohesive strength and stiffness:** Comparison of cohesive strength and stiffness of Resilon and gutta-percha has revealed that the cohesive strength (which is the tensile stress when they begin to flow or break) and modulus of elasticity (or stiffness) of gutta-percha and Resilon are relatively low with no clinically significant differences.^8^

**Radiopacity:** Both Resilon and gutta-percha have greater radiopacity values compared to dentin. Additionally, the mean radiopacity values in root canals were 5.50 and 8.52 mm of aluminum for Resilon and gutta-percha, respectively.^9^

### Mechanical and thermal properties

Tensile strength, elastic modulus and melting point of Resilon and gutta-percha are relatively the same; however, the endothermic enthalpy change and specific heat of gutta-percha are lower than those of Resilon.^10,11^ In addition, thermoplasticity of Resilon is higher than those of conventional and thermoplastic gutta-percha,^12^ such as Obturaflow, Endoflow and Regular Obtura.^13^

### Degree of conversion

Degree of conversion (DC) of a resin-based material refers to monomeric conversion of carbon-carbon double bonds to polymeric carbon-carbon single bonds. As a resin-based sealer, DC is one of the most important characteristics of Real Seal SE, influencing both biocompatibility and bonding. Biocompatibility of root canal sealers has major implications in the outcome of endodontic treatment. Freshly mixed and set conditions of Resilon/Epiphany have shown moderate to severe cytotoxic effects. One important reason is the leaching of uncured monomers that might induce deleterious effects on the periapical and periodontal tissues, such as inflammatory reactions, cytotoxicity, mutagenesis, and apoptosis. However, low DC impairs the bond of resin adhesive, which is important for maintaining the tight seal in the root canal filling. Moreover, DC is an important factor affecting setting shrinkage behavior of resin-based endodontic sealers. One study investigated the time-dependent change of DC of Real Seal SE as well as the influence of canal moisture and root canal depth on it. The results showed a significant increase in DC of Real Seal SE in 1 week, with stable DC after 1 week. Canal moisture decreases the DC but DC of Real Seal SE at different levels of tooth sections is not significantly different.^14^

### Bond strength of Resilon to root canal dentin

New technologies in endodontic obturation materials have addressed bonding by creating resin-based systems in combination with sealers that penetrate into and bond to the dentin wall and core obturation materials.^15^ Resilon as a new thermoplastic obturation material comes from the polycaprolactone polymer which has a low (62°C) glass transition temperature while its ability to bond with methacrylate-based resins is attributed to the fact that dimethacrylate monomers are blended into the polymer.^16^ The thermoplastic filling material is thought to be capable of bonding to a variety of dentin adhesives and resin cement-type sealers, an example of which is Epiphany. By combining self-etching adhesives and methacrylate-based resin sealers with Resilon, the manufacturer introduced what they termed a monoblock bonding concept for the obturation of root canals.^17^

Push-out bond strength of Resilon to root canal dentin, as a method of measuring bond strength, has been evaluated in many studies. These studies have compared Resilon with gutta-percha. Some of them have shown higher push-out bond strength for gutta-percha;^18-23^ in one of them there were no significant differences.^24^ It should be pointed out that in these studies gutta-percha and Resilon have been used with epoxy sealers and Epiphany. One of these studies showed that the bond strength of Epiphany + gutta-percha>AH-plus + gutta-percha>Epiphany + Resilon>AH-plus + Resilon.^21^ However, other researchers showed that the mean bond strength to root canal dentin was significantly higher in the Resilon/Epiphany group as compared to the gutta-percha/Kerr pulp canal sealer EWT group and gutta-percha/Roeko seal^25-27^ and bonding strength was greater in single-cone technique than thermoplastic obturation.^26^

Assessment of different irrigation protocols on push-out bond strength of root canal filling materials has shown that the bond strength of Resilon/Epiphany SE is not different after irrigation with 5.25% NaOCl, 2% CHX, 17% EDTA^28^ or 5.25% NaOCl + EDTA and 1.3% NaOCl + MTAD.^29^ However, one study showed that irrigation with 5.25% NaOCl + EDTA is a better conditioner than 1.3% NaOCl + MTAD for gutta-percha-AH26.^30^ It has been suggested that 5.25% NaOCl adversely affects the push-out bond strength of gutta-percha/AH-plus but CHX does not.^31^ Push-out bond strength of the Epiphany/Resilon obturation system with respect to different photo-activation methods has been examined in one investigation. The study has shown significantly higher bond strength values with the Quartz-Tungsten-Halogen and lowest push-out bond strength values with the plasma arc curing group.^32^ Other studies have shown that different light-emitting diode (LED) polymerization modes (20 seconds of maximum intensity versus exponential 5 seconds of exponential power increase, followed by 15 seconds of maximum intensity) have no significant advantage over the standard regimen in terms of dentin bond strength.^33^ Regarding bond strength after root canal retreatment using different solvents, it has been shown that Endosolve R has no significant influence on bond strength but chloroform has an adverse effect on the bond strength of Resilon/Epiphany SE after root canal re-obturation.^24^

It has been shown that Resilon with Hybrid root canal sealer results in greater push-out strength to root canal dentin compared to Epiphany SE and Epiphany.^34^ Another study has examined the push-out bond strength of the dentin-sealer interface with and without a main cone, showing that the epoxy resin-based sealer provides the highest push-out bond strength values. Push-out bond strengths were significantly higher when canals were filled with sealer alone than those filled with main cone and sealer using AH-plus, Endo REZ or Resilon sealers.^35^ One study evaluated shear bond strength of AH-26 and Epiphany sealers to both Resilon and composite resin. The results of the study showed significantly greater bond strength of AH-26 to both substrates compared to that of Epiphany.^36^ In another study the shear bond strength of Resilon to Real Seal was evaluated by using composite as the control group. The composite control exhibited mean shear strengths higher than those of Resilon.^4^

In conclusion, regarding bond strength, by considering the comparisons mentioned above, it seems that gutta-percha is superior to Resilon. In relation to factors like irrigation regimen, polymerization mode and bond strength of different sealers, there were few articles; therefore, further studies are necessary in order to evaluate their influence on bond strength.

### Leakage studies

Apical periodontitis is caused by intracanal bacteria. Prevention or healing of apical periodontitis involves a combination of disinfection of the root canal space through chemomechanical means and sealing of both the root canal and access cavity with materials that will prevent re-infection. Presently, the requirements for instrumentation of the root canal that will result in predictable success rates are well established. However, once the canal has been optimally disinfected, the present filling materials and techniques fail to achieve the requirement of providing a suitable seal to further challenge by bacteria.^37^ Therefore, some studies have evaluated sealing ability of Resilon using different methods that will be mentioned in the next paragraphs.

### Bacterial leakage studies

Two studies showed no statistically significant differences in resistance to leakage between Active GP/glass-ionomer sealer, Resilon/Epiphany, and gutta-percha/AH-plus.^1,38,39^ However, other investigations have indicated that some obturation systems like Guttaflow and Resilon cones with Epiphany provide better seal against microbial leakage than gutta-percha/AH-plus sealer.^40-43^, However, greater amount of microleakage has been reported with Resilon compared to gutta-percha/zinc oxide eugenol and AH-plus.^44,45^ It has been suggested that Resilon and MTA leak less than Super-EBA, with no differences between Resilon and MTA.^46^

In relation to the effect of obturation technique on leakage, no significant differences have been reported between lateral compaction with system B^38,47^ and lateral compaction with continuous wave compaction.^48^

Sealing ability of different sealers in comparison with Resilon ealers has been assessed. One study has evaluated the sealing ability of AH-plus, Epiphany, Acroseal, Endofill, and Polifil, showing the worst sealing ability for AH Plus and Endofill compared to Polifil, which showed the least leakage. Acroseal and Epiphany have shown a tendency toward an intermediate behavior, with no significant differences between Acroseal, Epiphany, and other sealers.^49^ Another study has assessed coronal bacterial leakage in root canals filled with new and conventional sealers, showing that Epiphany and Guttaflow with primer, along with Apexit, exhibit better resistance to bacterial penetration compared to AH-plus, RoekoSeal, EndoREZ and Acroseal.^50^ One study has shown that Epiphany takes more time to display microleakage than AH26 and AH-plus.^51^ Sealing ability of Epiphany was less than AH-plus and Seal Apex but better than MTA-based sealer and Active GP in one study.^52^

Regarding canal medication materials, it has been reported that calcium hydroxide dressing has no significant effect on sealing ability of Resilon.^53^

One study has focused on the effect of post space preparation on leakage, demonstrating no significant differences between immediate and delayed post space preparation using Resilon/Epiphany in relation to leakage.^54^

### Dye penetration studies

One study has shown that root canals filled with resin-coated gutta-percha/EndoRez and Resilon/Epiphany exhibit significantly less leakage than gutta-percha/Grossman’s sealer.^55^ Less dye penetration has been reported with Resilon/Epiphany compared to gutta-percha/AH-plus.^56-61^ Another study has shown that both materials provide the same coronal seal whereas Resilon provides the best apical seal.^62^ In contrast, another investigation has shown that the coronal sealing ability is the best for Resilon.^63^ A dye leakage test demonstrated that AH-plus, Epiphany and Sealapex permitted less leakage than Pulp Canal Sealer.^64^

No significant differences have been reported between gutta-percha and Resilon with the use of lateral condensation, hybrid technique and system B in relation to dye microleakage.^65,66^ However, one study has shown significantly more linear dye penetration than Resilon or gutta-percha subjected to lateral compaction with single-cone Resilon obturation technique.^66^

Regarding the relationship between canal irrigation solutions and sealing ability, it has been reported that CHX exhibits higher apical leakage values compared to MTAD and NaOCl in gutta-percha/AH-plus or MM-seal and Resilon/Epiphany SE, with the least leakage values in groups irrigated with MTAD irrigation solution. ^67^

### Marginal adaptation

In one study marginal adaptation of gutta-percha/AH26 and Resilon points/RealSeal was evaluated; the results showed that gaps were more frequent with gutta-percha, preventing hybridization with dentin.^68^ In another study the ultrastructural quality of the apical seal achieved with Resilon/Epiphany and gutta-percha/AH-plus were compared. The results revealed both gap-free regions and gap-containing regions in canals filled with both materials. It was concluded that a complete hermetic apical seal cannot be achieved with either of the two root canal filling materials.^69^ In yet another study interfacial quality was compared between Epiphany and Epiphany SE and the surrounding dentin with conventional gutta-percha/AH plus root filling as reference for comparison. The results showed that nonbonding AH-plus/gutta-percha root fillings exhibited a significantly higher amount of gap-free regions and displayed significantly narrower gaps compared with the two adhesive root-filling groups.^70^ Comparison of the adaptation quality of Guttaflow, Resilon/Epiphany and Endo REZ revealed no significant differences between the materials.^71^ It was reported that gutta-percha and Resilon-obturated canals subjected to occlusal load displayed significantly greater interface disruption compared with unloaded controls.^72^

### Leakage evaluation by fluid filtration technique

It has been suggested that there are no significant differences between sealing ability of Resilon/Epiphany and gutta-percha/AH-plus,^73-77^ and between gutta-percha/AH-plus and Resilon/Hybrid Root Seal or Real Seal.^78^ However, some studies have shown that their sealing ability is similar in the short term, with higher leakage with Resilon in the long term compared to gutta-percha.^79-81^ In contrast, in one study their leakage was not different over time.^73^ Another study showed greater leakage with Resilon regardless of time.^82^ Some investigations have revealed greater leakage of gutta-percha/AH26 compared to Resilon/Epiphany and gutta-percha/AH-plus.^75,83,84^ Some other studies have shown that gutta-percha/Sealapex has more leakage than Resilon/Epiphany.^85^ It has been reported that sealing ability of Real Seal is better than Termafill and One-step system.^86^Another study evaluated the sealing ability with different combinations of core and sealer and showed that MM-Seal/Herofill combination exhibited the least microleakage, and Real Seal/Herofill combination ranked second in this regard. The mean leakage values for the Real Seal/Resilon and MM-Seal/Resilon combinations were both significantly higher than the means for the other four experimental groups. Hybrid Root Seal combined with Resilon resulted in significantly less microleakage than Hybrid Root Seal combined with Herofill.^87^

Studies on the effect of irrigation solutions on leakage have shown that different final irrigation protocols with 5.25% NaOCl, 0.12% chlorhexidine, or 2% CHX and distilled water have no significant effect on leakage of Resilon.^88,89^A study showed that MTAD does not adversely affect the sealing ability of Resilon/Epiphany.^90^

In relation to the influence of calcium hydroxide on sealing ability one study investigated the long-term sealing ability of gutta-percha–Endofill and Resilon and Real Seal after calcium hydroxide dressing. Results indicated that groups with Ca(OH)_2_ dressing exhibited higher leakage values than groups in which Ca(OH)_2_ was not used.^91^ The effect of post space preparation on leakage was the other factor investigated in some studies. No differences were reported in microleakage between groups filled with gutta-percha/AH-Plus and Resilon/Epiphany after immediate preparation. However, Epiphany/Resilon obturation achieved better sealing in delayed post space preparation.^92^ In contrast, another study showed that immediate post space preparation is associated with less microleakage.^93^ The sealing properties of a one-step obturation post placement technique consisting of Resilon-capped fiber post obturators were compared with a two-step technique based on initial Resilon root filling followed by a 24-hour delay in fiber post placement. The results showed a significantly greater amount of fluid leakage with the one-step technique.^94^

The interrelationship between laser and curing mode with sealing ability has been addressed in a few studies and the results have been reported as follows. One study evaluated the sealing ability of Resilon by using different methods for curing Epiphany and reported the following statistical ranking for fluid filtration values: uncured, plasma arc curing, light-emitting diode, quartz-tungsten-halogen.^95^ One study evaluated the effect of Er,Cr:YSGG laser irradiation on the apical sealing ability of AH-plus/gutta-percha and Hybrid Root Seal/Resilon. The results showed a significant decrease in the microleakage values of all the experimental groups tested with time. EDTA + AH-plus/gutta-percha combination exhibited the least microleakage, whereas laser irradiation + Hybrid Root Seal/Resilon combination showed the greatest microleakage.^96^

Coronal seal has also been examined and it has been revealed that Epiphany/Resilon has higher leakage than specimens filled with AH-plus/gutta-percha, regardless of the coronal sealing condition and period of evaluation. No difference was detected between coronal restorative materials: Coltosol or Clearfil SE Bond.^97^ Another study has shown the same results by using glass-ionomer.^98^

Regarding the effect of different obturation techniques on leakage, it has been reported that Hybrid Root Seal has less fluid movement with cold lateral and vertical condensation techniques when compared with Thermafil and Ultrafil techniques.^99^ Another investigation has shown no significant differences between single cone, lateral compaction and system B techniques regarding sealing ability.^95^

### Glucose leakage studies

One study evaluated the reactivity of different endodontic materials and sealers with glucose in order to assess the reliability of the glucose leakage model in measuring penetration of glucose through these materials: Portland cement, MTA (grey and white), AH26, calcium sulfate, calcium hydroxide, AH26, Epiphany, Resilon, gutta-percha and dentin. The concentration of glucose was evaluated using an enzymatic reaction after 1 week. This assessment revealed that Portland cement, MTA, Ca(OH)_2_ and AH26 react with 0.2 mg mL^-1^ of glucose solution. Therefore, these materials should not be evaluated for sealing ability with the glucose leakage model.^100^

The results of one study showed that there is no significant difference between sealing ability of various combinations of Resilon, gutta-percha, AH-plus and Epiphany via fluid filtration method, with Resilon/AH-plus exhibiting significantly better sealing ability with the use of glucose technique.^101^ Another study clearly showed no significant differences in leakage between the two filling materials, Resilon with Epiphany sealer and gutta-percha with AH26 in both models.^102^ The results of another study showed the same results for AH-plus.^103^ It has been demonstrated that Resilon performs better than gutta-percha and MTA in the short term, but the seal of MTA and gutta-percha improve over time whereas the seal of Resilon deteriorates.^104^ The results of one investigation have suggested that glucose penetration is affected by the obturation technique. Ketac-Endo, either with gutta-percha or Resilon, exhibited significantly less glucose penetration in warm technique, whereas gutta-percha/Epiphany had significantly less glucose penetration in cold technique.^105^

In one study leakage was measured along root fillings with or without the smear layer using gutta-percha/AH26 and Resilon/Epiphany. The results revealed that glucose penetration model was more sensitive in detecting leakage along root fillings. Removing the smear layer before filling did not improve the sealing of the apical 4 mm of filling. Resilon allowed more glucose penetration but the same amount of fluid transport as the gutta-percha root fillings was observed.^106^

In conclusion, in glucose and marginal adaptation studies, in the majority of cases no significant differences have been detected between gutta-percha and Resilon. In bacterial and especially dye penetration studies Resilon has been a superior material in relation to sealing ability in comparison with gutta-percha, but in contrast, fluid filtration technique has shown that gutta-percha is superior to Resilon. On the whole, considering all the evaluation methods most of the investigations have indicated that Resilion is superior to gutta-percha in terms of sealing ability. Based on current studies it can be summarized that regarding post space preparation there is controversy over delayed and immediate preparation. In relation to obturation technique most studies have concluded that there are no significant differences between the methods mentioned. Further studies are necessary in order to reach a consensus over irrigation regimen and intracanal dressing.

### Clinical outcomes

Healing rates for Resilon-filled teeth in private practice have been reported to be within the range of success rates for studies with uniform treatment techniques mostly in university settings with gutta-percha root filling.^107^ A study showed that root canal systems obturated with gutta-percha and Kerr Pulp Canal Sealer or Resilon and Epiphany sealer have statistically indistinguishable differences in clinical outcomes.^108^ Finally, one report has claimed that resin-percha will successfully replace gutta-percha in near future.^109^ Finally, it can be concluded that the number of articles on this subject is too limited to consider Resilon a material superior to gutta-percha in terms of clinical outcome.

### Retreatment of root canals obturated with Resilon

Safe and efficient removal of filling materials from the root canal system is essential for optimal success. It has been shown that root canals filled with Resilon exhibit less remaining filling material after retreatment when compared with gutta-percha.^110-116^ However, one study showed that the difference was not significant.^117,118^ In contrast, another study showed better removal of gutta-percha.^119^ Resilon was removed faster than gutta-percha.^110,111,116,120^ But one study showed that there were no significant differences between gutta-percha and Resilon regarding the time needed for removal.^112,121^ In relation to the instruments used for the removal of filling materials, it has been shown that rotary files with chloroform are more effective than rotary files with heat,^110^ K3 than Liberator files, ^111^ Gates-Glidden than system B^116^ and heat than K3.^122^ There were no significant differences between Mtwo, Twisted files, Protaper and R-Endo^115^ and Hedestrome files in terms of Resilon removal.^118^ Resilon has higher solubility than gutta-percha in chloroform and its solubility increases over time.^123^ Another study has shown that citrol orange oil, eucalyptol and tetrachloroethylene are less effective on Resilon than on gutta-percha and xylol is more effective than orange oil and eucalyptol.^124^ No statistically significant effect of different obturation materials has been reported on the accuracy of Root ZX and ProPex for tolerance limits of 0.5 1.0 mm.^125^

Finally, based on data available, rotary instruments with suitable solvents such as chloroform can remove Resilon faster and better than gutta-percha. It is obvious that further studies are necessary to find better methods and solvents to remove root canal filling materials.

### Cytotoxicity

It has been shown that Resilon and gutta-percha have acceptable biocompatibility.^126-129^ Resilon has manifested less periradicular inflammation than gutta-percha.^2,130^ However, some studies have shown that Resilon is more cytotoxic than gutta-percha and its cytotoxicity increases after 48 hours.^131,132^ It has been suggested that cytotoxicity of Resilon + Epiphany, mainly due to Epiphany, decreases after 2 days to reach a level comparable to commonly used root canal sealers.^133^ In one investigation Epiphany was more cytotoxic than conventional materials^130^ but in another one they exhibited the same results.^134^ One study revealed that tissue tolerance of Real Seal cannot be attributed to its primer content.^135^

Therefore, it can be concluded that Resilon is a biocompatible material but there is still controversy over a change in cytotoxicity with time and further investigations are necessary.

### Obturation quality

Root canals filled with gutta-percha using lateral condensation technique exhibit more voids than those obturated with Resilon;^136,137^ however, continuous-wave compaction and vertical compaction has not resulted in any significant differences.^136,138^ Resilon flows better into lateral canals in single backfill technique.^139^ Resilon and Endo Flow gutta-percha have been effective in filling lateral canals by using the Obture II system compared to Obtura Gutta.^140^ One assessment indicated that there was no significant difference between root canals filled with Resilon and gutta-percha regarding percentage of canal space occupied by core material, sealer, voids and debris.^141^ One study has shown that Epiphany has significantly less percentage of sealer penetration in the apical thirds than the middle or coronal thirds;^142^ in addition, average penetration of Epiphany into dentinal tubules within the roots was deeper than those of Epiphany SE and AH-plus.^143,144^ In fact, by considering these few studies it is very difficult to conclude that Resilon is better than gutta-percha in terms of obturation quality, but it appears that the effect of obturation technique is important.

### Influence of Resilon on root fracture resistance

It has been reported that obturation of roots with resin-based filling materials, like Resilon, increases the resistance of roots to fracture when compared with gutta-percha.^145-148^ However, one study showed that forces to fracture were higher in root canals filled with AH-plus/gutta-percha.^149^ In contrast to these two groups of studies, some investigations have concluded that there is no significant improvement in resistance to vertical root fractures with the use of Resilon compared with conventional gutta-percha and sealer.^150-158^ One investigation demonstrated that fracture resistance of roots are significantly affected by the type of intra-orifice barrier, and the use of Vitremer and fiber-reinforced composite resin significantly improves fracture resistance; on the other hand, MTA has not exhibited any reinforcing effect as an intra-orifice barrier.^155^

Instrument separation often occurs during endodontic procedures and attempt to retrieve the separated segment usually contributes to dentin loss. It has been suggested that Resilon and MTA appear to compensate for root dentin loss that occurs as a consequence of attempts at retrieval of fractured instruments when used as canal filling materials but gutta-percha does not exhibit this effect.^159^

Regarding immature teeth, it has been reported that there is no significant difference between gutta-percha and Resilon regarding reinforcement of the roots of immature teeth.^160,161^ Root canals filled with hybrid composite resin (BisFil II) and Ribbond fibers with Panavia F luting cement have exhibited significantly more fracture resistance than Resilon.^160,162^ Other investigations have focused on the type of sealer, demonstrating that Hybrid Root Seal and iRootsp reinforced the immature roots against fracture when used with gutta-percha or Resilon but Endo REZ and AH-plus did not.^163^

In conclusion, it can be claimed that systems aiming at obtaining a monoblock system are not superior to gutta-percha in terms of fracture resistance of roots.

### Disinfection of Resilon

Any substance and material placed in the root canal either temporarily or permanently has shown the same result for Enterococcus faecalis and Candida albicans, demonstrating that 1% and 5% NaOCl solutions are effective agents for disinfecting Resilon cones in 1- or 5-minute treatments^167^ and 0.5-2.5% in 1 minute.^168^ Two-percent CHX was only effective after 5 minutes of treatment.^167^ Considering the importance of disinfection of root canal filling materials, some articles have been published on the effect of disinfecting materials on Resilon. It has been manifested that application of 5.25% NaOCl and 2% CHX increases surface free energy, prompting high interaction between Resilon and gutta-percha.^169^ It has been concluded that Resilon cones exposed to CHX for 10, 20 and 30 minutes demonstrate residual antibacterial action and that these agents do alter cone surfaces.^170^ Another study has shown the same results with one-minute immersion^165^ and with NaOCl for 30 minutes.^171^ By using scanning electron microscopy it has been shown that in the gutta-percha cones, NaOCl application for 10 minutes generates more irregular areas than CHX for 15 seconds and surface alteration is greater in gutta-percha than in Resilon.^172-174^ One study has shown that the final rinse is essential, especially when NaOCl and MTAD are used for canal disinfection because they cause crystal formation on Resilon cones but this change is not observed when 0.2% CHX is used.^175^ In general, considering these results it can be concluded that 1-minute 1% NaOCl is the best solution (like the routine method for gutta-percha) and time-consuming for disinfection of Resilon.

### Antimicrobial and antifungal activity of Resilon

One investigation manifested that Resilon cones exhibited no antimicrobial effect on Enterococcus faecalis, Pseudomonas aeruginosa, Porphyromonas endodontalis and Candida albicans except for Staphylococcus aureus. It showed antimicrobial efficacy against S. aureus during the first 24-hour period. However, after 48 and 72 hours, Resilon cones no longer inhibited the growth of S. aureus. In addition, gutta-percha and Resilon demonstrated no antifungal activity during any of the three test periods.^175^ Another study showed that Resilon does not exhibit antimicrobial properties against Actinomyces israelii, Actinomyces naeslundii, Enterococcus faecalis and Fusobacterium nucleatum.^176^ Therefore, it can be clearly concluded that Resilon has no superiority over gutta-percha regarding antibacterial properties against different microorganisms.

## Conclusions

Resilon like gutta-percha is a biocompatible filling material and it appears that the clinical outcome and obturation quality are similar, too. In term of bond strength it seems that gutta-percha is superior to Resilon; in contrast, sealing ability of Resilon, regardless of the evaluation method, is better than gutta-percha. Resilon has no superiority over the gutta-percha regarding reinforcing the obturated roots and antibacterial properties. However, more clinical studies are needed to confirm its efficacy compared with other materials.
